# Magnetic nanoparticle formulations to activate TREK and Piezo mechanosensitive ion channels (MICA) for enhanced osteogenesis

**DOI:** 10.1093/rb/rbag145

**Published:** 2026-07-15

**Authors:** Kritika, Michael Rotherham, Yuxuan Du, Kantawat Leerattanapetch, Thomas J Broomhall, Indrajit Roy, Alicia J El Haj

**Affiliations:** Healthcare Technologies Institute, Institute of Translation Medicine, School of Chemical Engineering, University of Birmingham, Birmingham B15 2TT, UK; Department of Chemistry, University of Delhi, Delhi 110007, India; Healthcare Technologies Institute, Institute of Translation Medicine, School of Chemical Engineering, University of Birmingham, Birmingham B15 2TT, UK; Healthcare Technologies Institute, Institute of Translation Medicine, School of Chemical Engineering, University of Birmingham, Birmingham B15 2TT, UK; Healthcare Technologies Institute, Institute of Translation Medicine, School of Chemical Engineering, University of Birmingham, Birmingham B15 2TT, UK; Healthcare Technologies Institute, Institute of Translation Medicine, School of Chemical Engineering, University of Birmingham, Birmingham B15 2TT, UK; Department of Chemistry, University of Delhi, Delhi 110007, India; Institute of Nano Medical Sciences, University of Delhi, Delhi 110007, India; Healthcare Technologies Institute, Institute of Translation Medicine, School of Chemical Engineering, University of Birmingham, Birmingham B15 2TT, UK

**Keywords:** magnetic nanoparticles, TREK antibody, Piezo antibody, osteogenesis, mechanotransduction

## Abstract

Magnetic nanoparticles (MNPs) have emerged as a potent tool in bone tissue engineering that utilize their intrinsic magnetism to influence the cellular microenvironment. Their capacity to boost osteoinductive, osteoconductive and angiogenic responses makes them exceptionally important for non-invasive regenerative therapies. This study presents a comparative analysis of synthesized uniform, small-sized (17–18 nm), spherical polyacrylic acid (PAA) functionalized iron oxide (IONPs) and cobalt ferrite (CFNPs) nanoparticles that were further functionalized with TREK and Piezo antibodies to precisely target these mechanosensitive ion channels. The osteogenic potential of these nanoparticles was assessed in MC3T3 and human adipose-derived stem cells under Magnetic ion channel activation (MICA) stimulation. Metabolic activity, cellular localization, ALP activity and alizarin red assay were assessed to get insights into cellular responses in osteogenesis. CFNPs exhibited comparable cellular uptake, increased metabolic activity, and elevated ALP levels relative to IONPs, especially at a low dosage of 5 µg. Alizarin red staining confirmed comparable mineralization in CFNP-treated groups under MICA stimulation compared to IONPs. Overall, these findings suggest that CFNPs, owing to high magnetic saturation, stable aqueous dispersion and a greater tendency to activate mechanosensitive ion channels than IONPs, offer a more potent platform for promoting osteogenic differentiation. This study highlights the potential for antibody-functionalized CFNPs combined with MICA as a promising non-invasive strategy for bone regeneration via mechanotransduction.

## Graphical Abstract

**Graphical Abstract:**
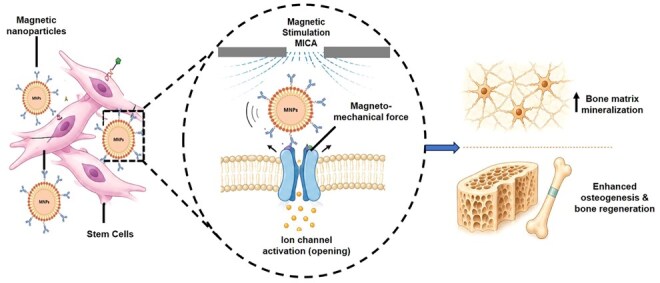

## Introduction 

Mesenchymal stromal cells (MSCs) have been demonstrated to have substantial therapeutic potential due to their capacity to differentiate into osteoblasts and mature into osteocytes, enabling bone remodeling and repair [1–3]. However, significantly large bone defects caused by traumatic accidents, degenerative illnesses, tumor resections, infections or congenital anomalies typically exceed the natural healing capacity of bone [4, 5]. Currently, bone autografts, allografts and metallic implants are common therapies for these abnormalities [6, 7]. However, their use is limited due to the involved surgical risks such as infection, bleeding and the challenges of harvesting large volumes of bone, which are required for autografts [8, 9]. Therefore, there is an immediate need for novel, non-invasive strategies that can overcome the critical bottlenecks of bone healing.

Magnetic nanoparticles (MNPs) made of metals, such as iron or cobalt, have emerged as promising tools in tissue engineering and regenerative medicine [10–12]. Their unique ability to react to applied magnetic fields allows a wide range of applications that rely on their sensitivity to external fields in both *in vitro* and *in vivo* conditions, such as magnetic hyperthermia, magnetic resonance imaging contrast agents and magnetically targeted delivery [3, 13–15]. In tissue engineering, MNPs provide remote activation of cellular pathways, enabling precise spatial and temporal control over cellular responses [13]. Previously, Kritika *et al.* have reported the successful synthesis and anticancer applications of uniform, small-sized (17–18 nm), spherical polyacrylic acid (PAA) functionalized iron oxide nanoparticles (IONPs) and cobalt ferrite nanoparticles (CFNPs). In the presence of a laser and magnetic field, these NPs were reported to demonstrate excellent biocompatibility, enhanced cellular uptake and tissue penetration in cancer therapy models [14, 16, 17].

Ion channel activation is essential for sustaining cellular functioning and homeostasis. Mechanosensitive (MS) ion channels embedded in the plasma membrane can convert mechanical stimuli into electrical or biochemical signals, also known as mechanotransduction, facilitating rapid cellular responses [18, 19]. TREK1 is a member of the two-pore potassium (K2P) family that is mechanoresponsive and responds to membrane stretch, change in temperature, pH and lipid interactions. Under mechanical stress, TREK’s transmembrane helices undergo rearrangement, stabilizing the open conformation and allowing K^+^ efflux. Besides the use of MNPs as anticancer agents, antibody-functionalized MNPs may be employed to bind specific cell membrane mechanoreceptors such as TREK1 for downstream cell regulation [13]. Dynamic magnetic field gradients induce torque on MNPs within the gradient, exerting mechanical stress on the attached cell surface mechanoreceptors [20, 21]. This stimulation activates mechanotransduction pathways and initiates specific cell signaling pathways [22, 23]. This mechanotransductive function is particularly important in bone homeostasis, cardiac physiology, and neuronal signaling, making TREK a potential target for regenerative therapies [13, 17, 23–25].

Piezo1 and Piezo2 are also MS ion channels [26]. The most well characterized of the Piezo family is Piezo1 (mPiezo1), which undergoes conformational changes and opens in response to increased membrane tension using a “force-from-lipid” mechanism [13, 27]. This structural shift widens the central pore, allowing cation influx (Ca^2+^, Na^+^, K^+^) and initiating downstream signaling pathways that regulate cellular functions. Moreover, Piezo1’s role in sensing mechanical forces in osteoblasts and osteocytes has been shown to be critical for bone homeostasis. Its deletion leads to reduced cortical thickness and cancellous bone volume, highlighting its potential as a therapeutic target for osteoporosis [28]. Piezo and TREK pathways could also act synergistically to enhance bone regeneration, offering a promising therapeutic method for bone regeneration.

While previous research demonstrated Magnetic ion channel activation (MICA)-mediated osteogenesis using large aggregate-type IONPs or graphene oxide-based magnetic systems, uniform, small-sized MNPs with distinct magnetic properties that affect MS ion channel activation and osteogenic differentiation are unexplored [13, 23]. Furthermore, no comparison studies on IONPs and CFNPs for selective activation of TREK1 and Piezo1 channels under MICA conditions have been reported.

**Figure 1:**
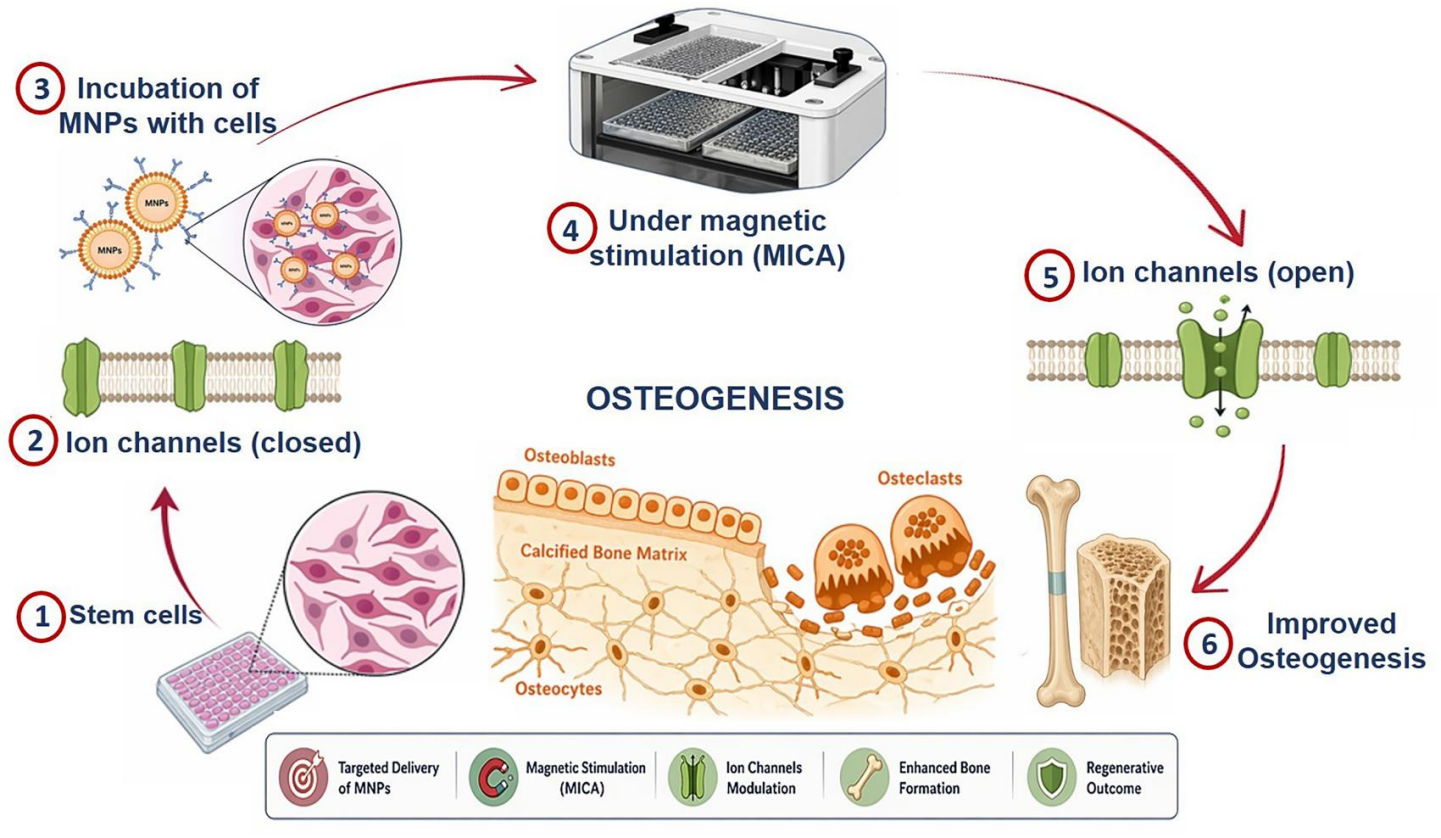
Schematic representation of antibody-functionalized MNPs incubated with stromal stem cells. Under magnetic stimulation (MICA), MNPs trigger ion channel opening thereby enhancing osteogenic differentiation and bone formation.

This study highlighted how superparamagnetic particles could be used and display ideal properties for magnetic cytoactivation. However, MNP aggregates are not uniform in shape and display variable sizes and properties, which is not ideal for clinical translation where regulatory bodies require tight release criteria on clinical products. To address this in our study, we have assessed the ability of functionalized IONPs and CFNPs with much more uniform sizes of 17–18 nm for activation using TREK and Piezo ion channels on osteoblast-like MC3T3 cells and human adipose-derived stem cells (hADSCs). Both particles were synthesized using the thermal decomposition method and coated with PAA to ensure water dispersion and provide carboxyl groups to allow functionalization with anti-TREK and Piezo antibodies that facilitate targeting to these MS ion channels. As PAA coating adds a thin, uniform layer around the MNPs, it retains the core magnetic properties more efficiently than bulkier coatings, which can reduce magnetic responsiveness and enable a smaller particle size to be used for our studies [29–31]. MNP retention plays a crucial role, particularly in magnetically guided bone regeneration therapies, which require precise and effective remote stimulation of MS pathways. The overall concept of this study is illustrated in Figure 1. Under MICA stimulation, the antibody-functionalized MNPs exert mechanical forces that activate TREK and Piezo channels, which thereby initiate intracellular signaling pathways that promote osteogenic differentiation and bone regeneration.

In this study, we synthesized and compared IONPs with novel CFNPs. We first characterized the MNPs using scanning electron microscopy (SEM)/EDAX analysis. Next we established the biological response of bone cells to the MNPs by testing metabolic activity and cellular localization to elucidate the optimal dosage, binding efficacy and cellular uptake of MNPs. We then compared the potential of these MNPs to magnetically stimulate MS TREK and Piezo ion channels to induce osteogenic differentiation and bone regeneration through ALP activity and calcium mineralization assays over 21 days.

## Materials and methods

### Synthesis of MNPs with antibody functionalization

**Figure 2:**
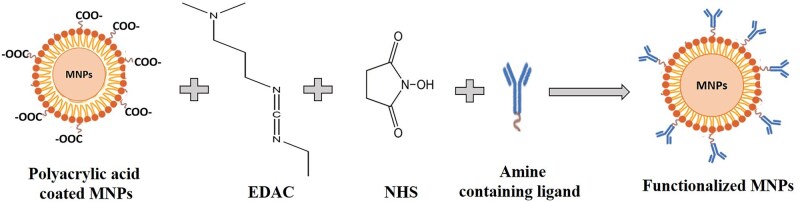
Systematic representation of functionalization of antibody on PAA-coated MNPs using EDAC chemistry.

The synthesis of PAA-functionalized IONPs and CFNPs was performed using the thermal decomposition method, followed by a ligand exchange process as previously described by Kritika *et al.* [14]. Briefly, cobalt acetate and iron acetylacetonate were heated at 295–310°C in high-boiling solvents, including oleic acid, oleylamine and 1-octadecene, and subsequently washed using hexane and ethanol. Ligand exchange method is used to functionalize the MNPs with PAA, followed by hexane–methanol (1:1) washing. The synthesized particles were characterized using transmission electron microscopy (TEM), dynamic light scattering (DLS), X-ray diffraction (XRD), fourier transform infrared spectroscopy (FTIR) and vibrating sample magnetometer (VSM). The MNPs were then functionalized with TREK antibody (Alomone Labs, APC-047, Israel) and Piezo antibody (Proteintech, UK) via carbodiimide chemistry in accordance with a previously reported protocol (Figure 2) [23]. Briefly, the MNP surface was activated by mixing in sterile 1-ethyl-3-(3-dimethylaminopropyl)-carbodiimide hydrochloride and N-hydroxysuccinimide in 0.5 M (2-(N-morpholino)ethanesulfonic acid) MES buffer (pH = 6.3) for 1 h at room temperature. The activated MNPs were washed and recovered using magnetic separation and washed with 0.1 M MES buffer. For antibody labeling, the MNPs (1 mg) were conjugated overnight or for 3 h with 20 µg of anti-rabbit secondary antibody (Abcam, Cambridge, UK). Particles were then washed in 0.1 M MES buffer, then coated with either 10 µg of TREK1-Ab (Alomone Labs, Jerusalem, Israel, http://www.alomone.com) or 10 µg of Piezo antibody (Proteintech, Manchester, UK) by mixing with 1 mL of 0.1 M MES buffer. The coated MNPs were then incubated with 10 µL of 20 mM glycine for 30 min at room temperature. Finally, the MNPs were washed in phosphate-buffered saline (PBS) and then re-suspended in 1 mL PBS. These antibody-functionalized nanoparticles are referred to as TREK-IONPs, TREK-CFNPs, Piezo-IONPs and Piezo-CFNPs, respectively. For MNP-antibody titration assays, commercially available superpara-MNPs, 250 nm in diameter, with surface carboxyl-coated dextran (Nanomag®, Micromod, Rostock, Germany) were coated with varying amounts of anti-rabbit secondary antibody and TREK1-Ab using the carbodiimide coating reaction as described above. Goat anti-rabbit antibody IgG Fc (ab97196; Abcam, Cambridge, UK) was added at concentrations of 0, 10 or 20 μg/mL, and TREK1 antibody was added at concentrations of 0, 5 or 10 μg/mL. The functionalized MNP suspension was then subjected to a final wash with 0.1 mL of 0.1% bovine serum albumin (BSA) (Bioreagent grade, Fisher, Loughborough, UK) and resuspended in 1 mL of 0.1% BSA, resulting in a final concentration of 1 mg/mL of anti-TREK1 functionalized MNPs with defined antibody loading ratios as shown in Supplementary Table S1.

### MNP characterizations

The detailed characterizations of PAA-functionalized MNPs were previously reported in Kritika *et al*. [14]. For TEM analysis, Thermo-FEI TECHNAI G2-T30, U-TWIN TEM system was used to characterize the size and morphology of MNPs. Briefly, formvar-coated copper grids were used, and the sample was deposited using the drop-casting method with drying at room temperature. The zeta potential analysis was carried out using a Malvern Zetasizer, 0.1 mg/mL solution of MNPs was sonicated for 5–10 min and 750 µL was transferred to a disposable folded capillary cells (DTS1070) for analysis. The magnetic characteristics were analyzed with a MicroSense EV-7 VSM.

### Cell culture

MC3T3 cells were cultured in α-MEM media supplemented with 1% penicillin-streptomycin and 1% L-glutamine and 10% fetal bovine serum (FBS). hADSCs were patient-derived cells obtained from the Royal Orthopaedic Hospital (ROH), Birmingham, UK, under application reference ROH19002. The ROH is an ethically approved tissue bank (East Midlands—Derby Research Ethics Committee, Ref: 17/EM/0030). The cells were cultured in Dulbecco’s Modified Eagle’s Medium (DMEM) (with L-Glu) supplemented with 10% FBS and 1% penicillin-streptomycin. The cultures were sustained at 37°C in a 5% CO_2_ atmosphere, with the media being changed twice a week.

For osteogenesis experiments, osteogenic media (OM) were made with basal media/α-MEM above with the addition of 0.2 mM ascorbic acid, 10 mM sodium β-glycerophosphate, and 0.1 μM dexamethasone (Sigma). The OM was freshly prepared and replaced every 2–3 days to facilitate optimal differentiation conditions.

HEK293-ERK luciferase reporter cell culture: HEK293-ERK luciferase reporter cells, also referred to as the SRE-Luciferase reporter HEK293 recombinant cell line, were obtained from BPS Bioscience, San Diego, CA, USA (catalog number: 60406). For expansion, cells were cultured in T25 flasks (Corning®, Oneonta, NY, USA) containing serum basal medium consisting of DMEM (Gibco, Thermo Fisher Scientific, Scotland, UK), supplemented with 10% FBS (Gibco, Thermo Fisher Scientific, Scotland, UK). Cells were expanded and passaged up to seven times prior to their use in labeling with MNPs.

#### SEM and energy dispersive X-ray analysis

The dose–response relationship between cell-MNP loading for samples treated with different doses (5 and 10 µg) after 72 h of incubation was assessed using a TM3030 SEM with energy dispersive X-ray (EDX) capabilities (Hitachi, Japan). After the incubation period, the treated hADSCs were fixated with 4% paraformaldehyde (PFA) in PBS to preserve cell morphology. The samples were dehydrated with ethanol and sputter-coated with a thin conductive gold to enhance surface conductivity. The samples were then analyzed under different magnifications to examine morphological alterations and nanoparticle distribution on or within the cells. EDX spectra were obtained to ascertain the elemental composition and to identify the presence of iron and/or cobalt signals, therefore validating MNP internalization or surface association.

#### Iron release quantification by inductively coupled plasma-optical emission spectroscopy

Iron release was quantified using MNPs with and without antibody coating in PBS using the PerkinElmer Optima 8000 inductively coupled plasma-optical emission spectroscopy (ICP-OES) instrument, following the established degradation protocol. Briefly, 2.5 µg/cm^2^ of IONPs, TREK-IONP, CFNPs, and TREK-CFNP were incubated in PBS (pH 7.4) for 24 h at 37°C. Following incubation, samples were centrifuged and filtered to remove residual nanoparticles, and Fe concentration in the supernatant was quantified by ICP-OES. The PBS control was used for background correction, and the results were expressed as PBS blank-corrected Fe concentration in ppb.

#### Cellular localization studies

Prussian blue staining was utilized as a qualitative histochemical technique to examine the cellular binding and localization efficiency of TREK- and Piezo-functionalized MNPs. This assay yields a characteristic blue coloration when ferric ions react with potassium ferrocyanide, hence validating the association of nanoparticles with the cells. Briefly, 1.5 × 10³ MC3T3 cells and hADSCs were seeded in a 96-well plate containing growth media and incubated overnight at 37°C in a humidified environment with 5% CO_2_ to facilitate cell adhesion. Following a 24-h period, the culture media were substituted with a fresh medium incorporating TREK- and Piezo-functionalized MNPs at final concentrations of 5, 10, and 25 µg per well. To enable intracellular uptake of the functionalized MNPs targeting the intracellular loop region of the TREK ion channel, DOTAP (N-(1-(2,3-dioleoyloxy)propyl)-N,N,N-trimethylammonium chloride) was employed as an internalization agent. DOTAP was added to each anti-TREK-functionalized MNPs condition at a ratio of 40 ng DOTAP per 1 mg of MNPs, vortexed and incubated at room temperature for 15 min to allow MNP-DOTAP partial complex formation. The cells were treated with nanoparticles for 1.5 h to enhance cellular absorption, subsequently followed by gentle washing with PBS to eliminate unbound or loosely attached particles.

After 72 h of post-treatment growth, the cells were fixed and stained with the Prussian blue reagent, which was made as a 1:1 mixture of 20% hydrochloric acid (HCl) and 10% potassium ferrocyanide aqueous solutions. The staining reaction was conducted for 20 min at ambient temperature, facilitating the production of blue ferric ferrocyanide complexes that signify intracellular iron. Thereafter, the samples were washed twice with distilled water and counterstained with Nuclear Fast Red for 5 min to see the cell nuclei. The stained cells were washed with distilled water and were imaged using a light microscope to qualitatively evaluate nanoparticle distribution and cellular localization.

#### HEK293-ERK cell labeling with functionalized MNPs

Labeling of the anti-TREK-functionalized MNPs to HEK293-ERK luciferase reporter cells was performed by seeding 1 × 10^5^ HEK293 cells per well in 24-well culture plates, followed by the addition of 0.5 mL serum-free basal DMEM. The cells were incubated for 3 h at 37°C with 5% CO_2_ to reduce interference from membrane-associated serum proteins and facilitate nanoparticle binding. To enable intracellular uptake, 6-μL aliquot of the DOTAP-MNP complex was then added to each well and incubated at 37°C with 5% CO_2_ for 1.5 h. The experiment was performed in triplicate for each condition of anti-TREK MNP coating concentration. Following incubation, cells were gently washed with DPBS, and the medium was replaced with 0.3 mL of reduced serum basal medium (DMEM supplemented with 0.5% FBS). The plates were then transferred to a magnetic force bioreactor for 1 h of magnetic force stimulation as described in the “Live/dead staining” section.

#### Luciferase reporter assay

Luciferase activity was quantified in HEK293-ERK reporter cells following 24-h exposure to anti-TREK functionalized Micromod MNPs with or without magnetic stimulation, using a luminescence-based reporter assay. At the end of the intervention, media from each triplicate condition were mixed 1:1 with luciferase reagent (ONE-Step™ Luciferase Assay System, BPS Bioscience, San Diego, CA, USA), prepared according to the manufacturer’s protocol, and incubated for 15 min. Cell-free wells containing only serum basal medium served as blanks. Luminescence from each condition, with or without magnetic field exposure, was measured at room temperature with a 3-s integration time using a SparkTM 10 M multimode microplate reader (TECAN, Männedorf, Switzerland) with a 24-well flat transparent plate (Cellstar®, Greiner Bio-One, Kremsmünster, Austria), as described previously (Unnithan *et al*., 2022). Fold induction of luciferase expression was calculated by dividing the background-subtracted luminescence of magnetic field-exposed samples by the average background-subtracted luminescence of unexposed samples.

#### dsDNA quantification for data normalization

Variation in cell number across samples was normalized using dsDNA quantification, which correlates with the number of cells in each well and allows for standardization across samples. The dsDNA quantification was performed following the luciferase assay using the Quant-iT™ PicoGreen™ dsDNA Assay Kits (Thermo Fisher Scientific, Eugene, Oregon, USA). The sample media were transferred to a 96-well black plate (Costar®, Kennebunk, ME, USA) and diluted 1:100 in TE buffer (10 mM Tris-HCl, 1 mM EDTA, pH 7.5). PicoGreen reagent, prepared according to the manufacturer’s instructions, was added, mixed thoroughly, and incubated at room temperature for 5 min. Fluorescence was measured using a Spark™ 10M multimode microplate reader (TECAN, Männedorf, Switzerland) at an excitation wavelength of 480 nm and emission of 520 nm. A dsDNA standard curve was performed according to the manufacturer’s instructions. The concentration of dsDNA was calculated from the standard curve and used for luciferase data normalization by dividing the fold induction by the corresponding dsDNA value.

#### Metabolic activity assay—PrestoBlue

The metabolic activity of the cells treated with TREK- and Piezo-functionalized MNPs was evaluated using the PrestoBlue cell viability assay. The MC3T3 cells and hADSCs were seeded at 1 × 10^4^ cells per well in a 96-well plate and incubated overnight under standard culture conditions (37°C, 5% CO_2_ humidified atmosphere). After 24 h of incubation, the culture media were replaced with fresh media, and different doses (5, 10, 25 µg) of TREK- and Piezo-functionalized MNPs were introduced into the wells. Following 90 min of incubation, the samples were carefully washed twice with PBS to remove excess unbound MNPs. Fresh media were then added, and the cells were further incubated for 72 h to facilitate nanoparticle–cell interactions. Thereafter, PrestoBlue reagent was added to each well and incubated for 10 min at 37°C. The fluorescence intensity was then measured on a SPARK Tecan plate reader using an excitation wavelength of 580 nm and an emission wavelength of 590 nm.

#### Live/dead staining

Live/dead staining (Invitrogen, Thermo Fisher) was used to qualitatively assess the cell viability of TREK- and Piezo-functionalized MNPs. The staining solution was made by combining 5 µL of calcein AM (Component A) and 20 µL of ethidium homodimer-1 (Component B) with 10 mL of DPBS. Approximately 15 × 10^3^ MC3T3 and hADSCs per well were seeded in a 96-well plate containing growth media. The cells were incubated overnight at 37°C in a humidified environment containing 5% CO_2_. Following 24 h of incubation, 5, 10 and 25 µg of TREK- and Piezo-functionalized MNPs were added to the wells and incubated for 1.5 h. Then the cells were washed with PBS to eliminate excess NPs, and fresh media were added.

After 72 h of growth, the cells were stained with live/dead staining to determine the viability. The staining solution was added to each well following the removal of the culture media. Then the cells were incubated for 30 min at room temperature (20–25°C) and subsequently imaged under an EVOS M5000 fluorescence microscope.

#### Dynamic magnetic field gradients

Cell samples were loaded into a magnetic force bioreactor (MICA Biosystems) and exposed to cyclic magnetic fields (≤250 mT) with a field gradient of 11 T/m, which was housed in a cell culture incubator (37°C, 5% CO_2_). Magnetic stimulation was applied at a frequency of 1 Hz in 5 × 1 h sessions per week [13].

#### Alkaline phosphate activity

Alkaline phosphatase (ALP) is an early-stage osteogenic biomarker used to assess the osteogenic differentiation of bone cells. Therefore, to evaluate the osteogenic differentiation potential of TREK- and Piezo-functionalized MNPs, MC3T3 cells (4 × 10^4^) and hADSCs (4 × 10^4^) were seeded in 24-well plates with growth media. After 24 h of incubation, TREK-functionalized MNPs were tagged to MC3T3 cells, while Piezo-functionalized MNPs were tagged to hADSCs at varying concentrations of 5, 10 and 25 µg. The OM was subsequently added to each well. The cells were then magnetically stimulated (≤250 mT) for an hour daily, with media replacements every 2 days.

The Sensolyte ALP test kit was used to quantify the enzymatic activity of ALP. ALP activity was evaluated in MC3T3 cells treated with TREK-functionalized MNPs and in hADSCs treated with Piezo-functionalized MNPs after 7 and 14 days of treatment. At each time point, cells were lysed with 0.1% Triton X-100, gently scraped, then centrifuged at 2500×g for 10 min to separate the enzymatic supernatant from insoluble cell debris. A 50-µL aliquot of the supernatant was combined with 50 µL of p-nitrophenyl phosphate solution in a 96-well plate and gently shaken for 30 s. The absorbance was then measured at 405 nm using a microplate reader after 1 h of incubation. Moreover, a standard curve was prepared from known protein (ALP) concentrations to calculate the concentration of ALP in the samples.

Additionally, the BCA Protein Assay Kit (Thermo Scientific, USA) was used to evaluate the total protein concentration in the samples. Briefly, the samples were mixed with BCA working reagent according to the manufacturer’s instructions and incubated at 37°C for 30 min. Then the absorbance was measured at 562 nm, and protein concentrations were calculated using a standard curve generated with BSA. The ALP activity for each sample was normalized to the total protein content and represented as units of ALP activity per mg of protein (U/mg protein) to account for variations in cell density among samples.

#### Alizarin Red staining

Calcium deposition in samples with and without MICA treatment was assessed using Alizarin Red staining (ARS, Alizarin-Red Staining Solution, Sigma Aldrich) after 21 days of culture. The media were removed, and the cells were gently washed twice with PBS. Subsequently, the cells were fixed with 4% PFA, and ARS solution was added to each well. After 10 min, the staining solution was removed and the cells were washed multiple times with distilled water until the rinse was clear. The stained cultures were examined under an optical microscope to observe calcium deposition.

For quantitative analysis, cetylpyridinium chloride (CPC) was used to quantify mineralization in the cells. Briefly, 500 µL of 10% CPC in 10 mM PBS (pH 7) was added to the samples and incubated at 37°C for 30 min. After 100 µL of the dissolved stain was transferred to a 96-well plate, the absorbance was measured at 562 nm with a plate reader.

### Statistical analysis

All experiments were carried out in triplicates unless otherwise indicated. Data analysis was done using the Origin statistical software (OriginLab Corporation). The results were presented as mean ± standard deviation, *n* = 3 (technical replicates). Statistical comparisons between two groups were carried out using Student’s *t*-test, while one-way or two-way analysis of variance was used for multiple group comparisons with post hoc Tukey tests. Statistically significant differences were indicated with ** for *P* < 0.01, *** for *P* < 0.001, **** for *P* < 0.0001.

## Results

### Synthesis and characterizations

**Figure 3:**
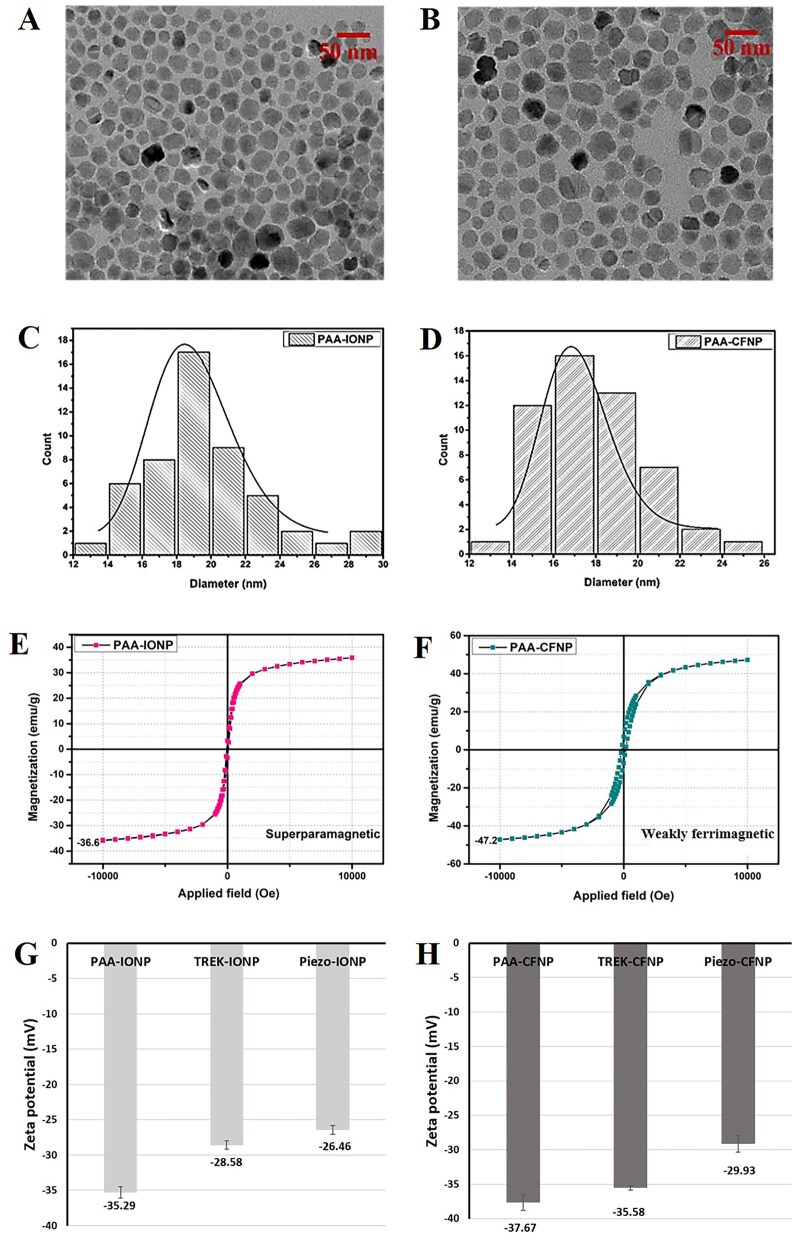
TEM images with histogram distribution plot showing average particle size of (**A**, **C**) 18-nm PAA-IONPs and (**B**, **D**) 17-nm PAA-CFNPs. Magnetic hysteresis measurements using VSM of (**E**) PAA-IONPs and (**F**) PAA-CFNPs. Zeta potential measurement of (**G**) PAA-, TREK-, Piezo-functionalized IONPs and (**H**) PAA-, TREK-, Piezo-functionalized CFNPs.

The physicochemical characteristics of PAA-IONPs and PAA-CFNPs demonstrated their potential for use in remote cell activation. Oleic acid-coated IONPs and CFNPs were synthesized using the thermal decomposition method followed by PAA functionalization via ligand exchange to enable aqueous dispersion and bioconjugation [14, 32]. The TEM images revealed the spherical morphology of both PAA-CFNP and PAA-IONP with average particle sizes of 17 and 18 nm, respectively (Figure 3A–D). These dimensions are within the ideal range (10–30 nm) for biomedical applications, as they offer properties such as physiological stability and biocompatibility [33]. The VSM suggests the ferrimagnetic and superparamagnetic behavior with magnetic saturation of 47.2 and 36.6 emu/g for PAA-functionalized CFNPs and IONPs, respectively (Figure 3E and F). The increased magnetic saturation highlights the superior magnetic properties of CFNPs, which play an important role in applications that require effective magnetic force for transduction in bone tissue engineering.

As reported earlier, the unfunctionalized PAA-IONPs and PAA-CFNPs exhibited notably high negative surface charges (−35.29 mV, −37.69 mV) due to the presence of carboxyl groups on PAA [14]. In this study, after coupling with TREK- and Piezo-antibodies, a significant shift toward less negative surface charge was observed, indicating successful antibody coupling via carbodiimide chemistry (EDAC-mediated coupling) (Figure 3G and H). Moreover, this alteration in surface charge showed the neutralizing effect of protein/antibody adhesion on the NP surface and confirmed the successful bioconjugation of the PAA-MNPs.

### SEM and EDX analysis

**Figure 4:**
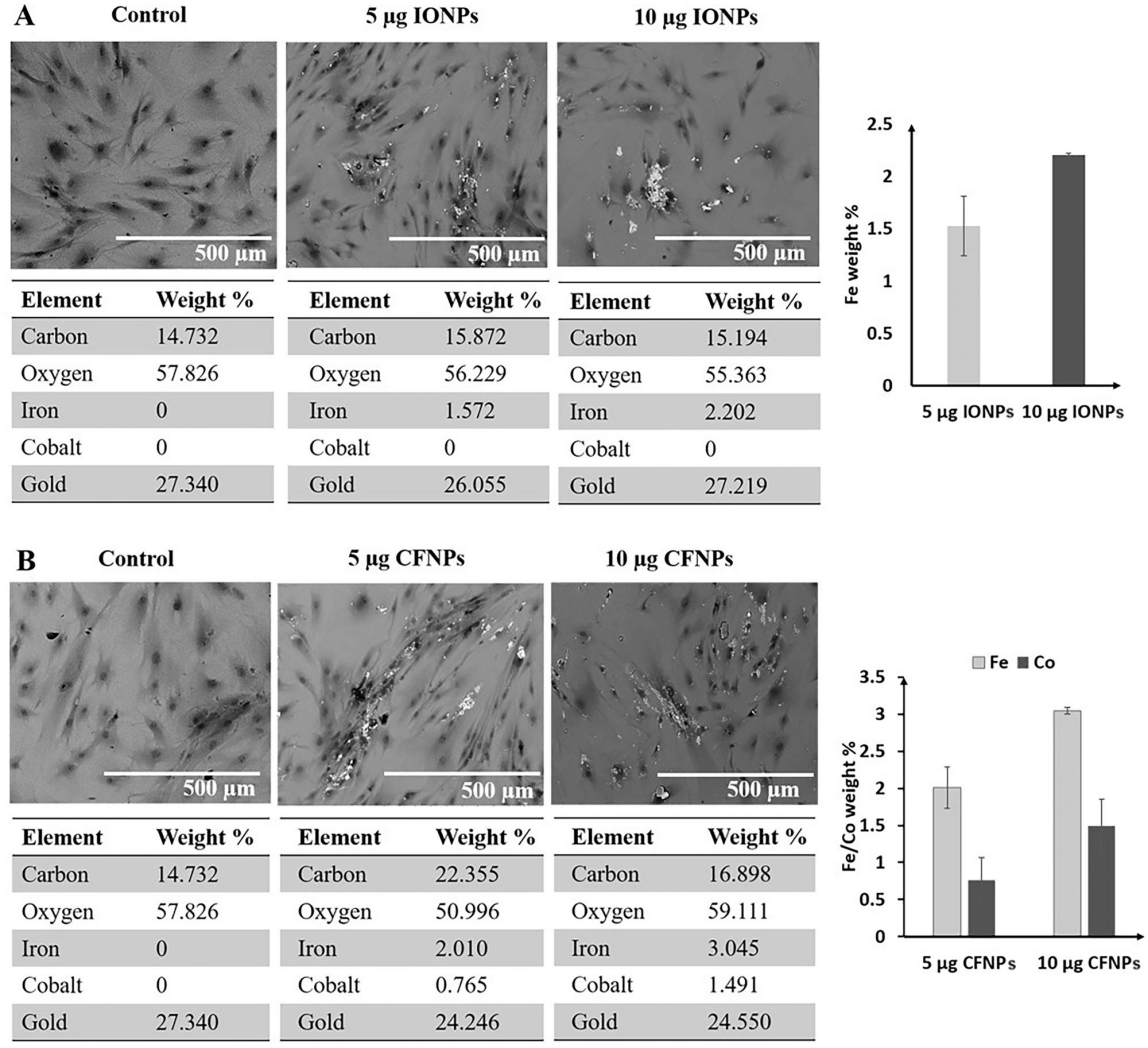
SEM with EDAX analysis after 72 h of treatment with (**A**) PAA-IONPs and (**B**) PAA-CFNPs using hADSCs (scale bar 500 µm).

The SEM and EDX analyses illustrate the dose–response relationship between cell-MNP loading for samples treated with different concentrations of MNPs (5 and 10 µg) after 72 h of treatment, as shown in Figure 4. In the control group (without MNPs), the cells maintained typical morphology. However, as the concentration of MNPs increased, visible clustering of MNPs around the cells was observed. At 5 µg, MNPs demonstrated effective cellular binding with minimal clustering, indicating stable dispersion and biocompatibility. Interestingly, this observation aligns with previous studies investigating MNP-mediated mechanotransduction, which used a comparable dose for initiating osteogenesis but with a different size of particle [3]. In contrast, at a 10-µg dose of MNPs, denser agglomerates were observed near the cells, which could potentially hinder downstream analysis. These findings suggested that a concentration of 5 µg is optimal for subsequent *in vitro* mechanotransduction and osteogenic investigations, providing a balance between efficient nanoparticle absorption and minimal morphological alteration. EDX analysis also supported the presence of iron and cobalt within the cells, substantiating nanoparticle internalization at this optimal dosage.

### Iron release quantification by ICP-OES

To assess the stability and degradation properties of functionalized MNPs (2.5 µg/cm^2^), iron release into PBS (pH 7.4) was measured by ICP-OES. After 24-h incubation at 37°C (Supplementary Figure S3), a comparatively higher Fe release was observed for IONPs than the TREK-IONPs. Whereas CFNPs and TREK-CFNPs have comparable values with TREK-CFNPs slightly lower Fe release. These findings indicate that surface functionalization, particularly antibody (TREK) coating, enhances nanoparticle stability by limiting iron dissolution under physiological conditions. The reduced Fe release suggests that the antibody layer acts as a protective barrier, reducing nanoparticle degradation. This property is advantageous from a biocompatibility perspective, as it may minimize excess iron-mediated cytotoxicity and oxidative stress, thereby supporting safer biological applications of the functionalized MNPs.

### Cellular localization studies

**Figure 5:**
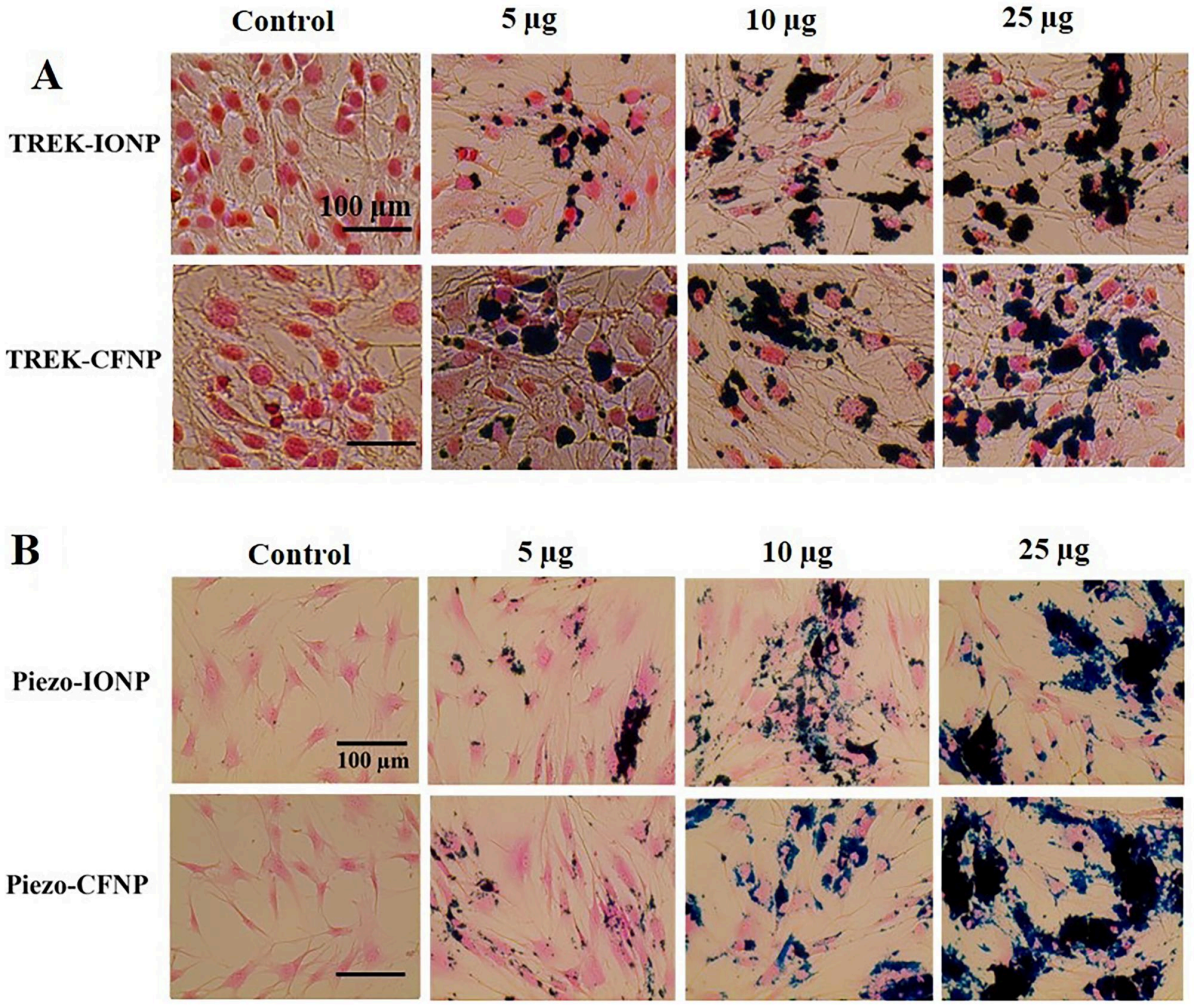
Concentration-dependent cellular localization after 72 h of treatment following Prussian blue staining of (**A**) TREK-IONPs and TREK-CFNPs in MC3T3 cells. (**B**) Piezo-IONP and Piezo-CFNPs in hADSCs (scale bar 100 µm).

The Prussian blue staining was used to evaluate concentration-dependent uptake of the TREK- and Piezo-functionalized MNPs in MC3T3 cells and hADSCs, respectively, after 72 h of treatment with 5, 10, and 25 µg doses (Figure 5). In both cell types, control groups (cells only) displayed negligible blue staining, indicating no iron accumulation. While all treated samples showed a dose-dependent increase in blue intensity, demonstrating effective cellular localization. Notably, at a lower dose (5 µg), MC3T3 cells exhibited higher blue staining intensity than hADSCs, indicating higher binding properties possibly due to their differentiated state, whereas hADSCs showed a more gradual uptake trend. At higher doses (10–25 µg), both cell types showed increased iron accumulation with localized aggregation, particularly in hADSCs, indicating higher sensitivity. Also, at the same dose, CFNP-treated cells showed higher blue intensity of staining in the cells as compared to IONPs in both cell types, suggesting enhanced cellular binding likely due to inherent high magnetic anisotropy and better dispersion of the CFNPs.

Nuclear fast red counterstaining revealed well-maintained cellular morphology in all treated groups, suggesting maintained cell viability. Thus, these studies combined with SEM/EDX data confirmed 5 µg as the optimal dose, allowing for effective cellular binding and high cell viability.

### Luciferase reporter assay

For antibody titration assays, an HEK293-ERK luciferase reporter cell line was labeled with commercial Micromod MNPs functionalized with varying amounts of anti-rabbit and anti-TREK antibodies as outlined in Supplementary Table S1, with or without magnetic stimulation. Luciferase activity was used as a readout for cellular ERK pathway activation in response to MNP and magnetic stimulation. Results showed that the highest induction in ERK pathway activation was achieved using a 20:10 μg ratio of secondary–primary antibody per mg of MNP (condition T1–9), which induced a statistically significant 1.857 ± 0.40-fold induction in luciferase activity (Supplementary Figure S1). While other secondary antibody concentrations did not reach statistical significance, fold induction tended to increase overall with higher secondary antibody concentrations in combination with the primary antibody. In addition, since the presence of TREK antibody had a significant effect on fold induction, a titration assay was conducted to determine which primary antibody concentrations significantly promoted ERK activation. Comparisons across three concentrations (0, 5 and 10 μg/mL of TREK) revealed significant increases in fold induction at both 5 μg/mL (*P *= 0.046) and 10 μg/mL (*P *= 0.013) (Supplementary Figure S2). This indicates that ERK pathway activation can be effectively achieved using a TREK primary antibody concentration of 5 μg/mL, with more robust activation achieved when using 10 μg/mL of antibody on the MNP surface.

### Cell viability assay

**Figure 6:**
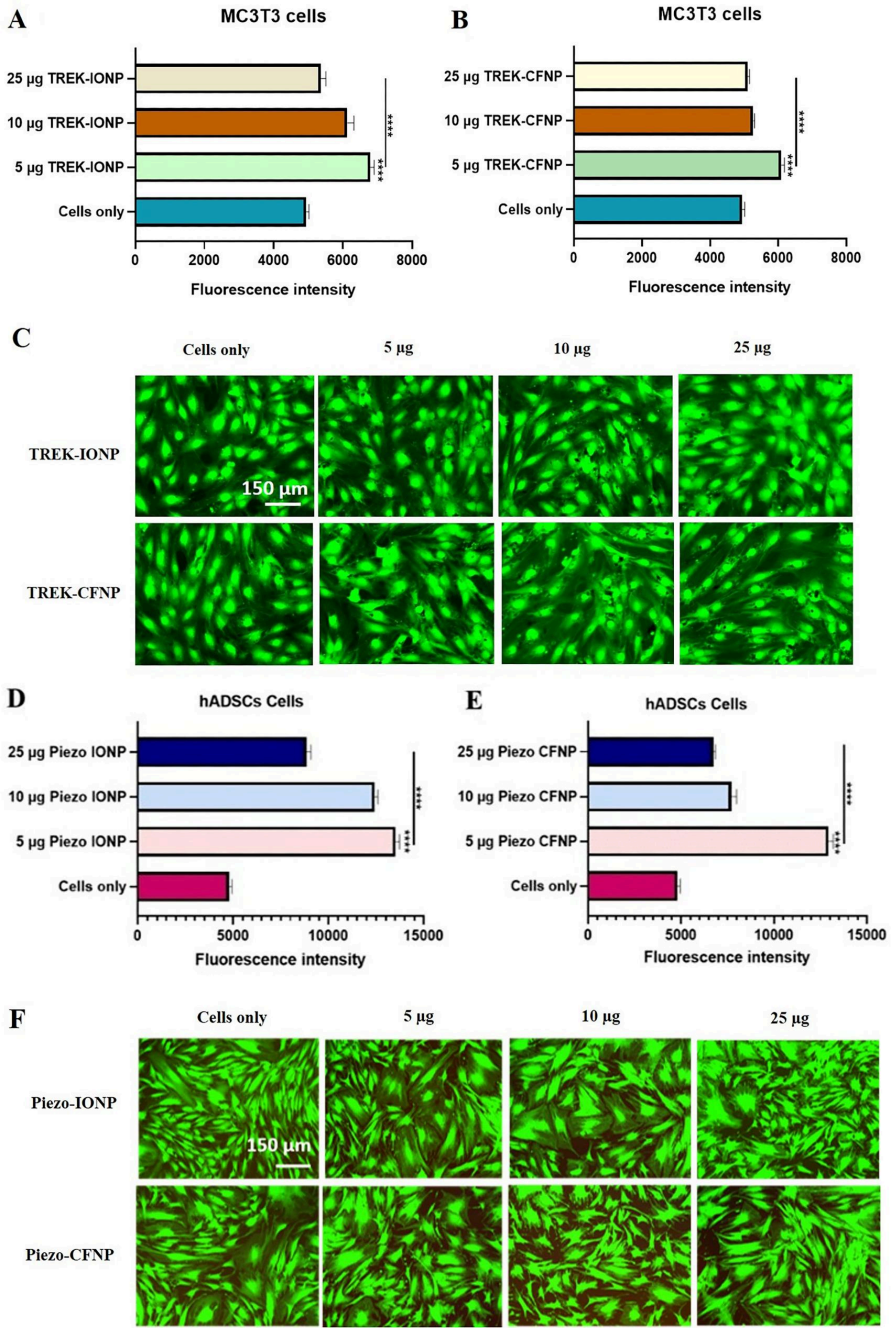
Concentration-dependent PrestoBlue assay after 72 h with (**A**) TREK-IONP, (**B**) TREK-CFNP on MC3T3 cells and (**D**) Piezo-IONP, (**E**) Piezo-CFNPs on hADSCs. Live/dead staining demonstrating cell viability after 72 h of treatment with MNPs on (**C**) MC3T3 cells and (**F**) hADSCs. Statistical comparisons in the PrestoBlue assay were performed both between treated groups and untreated control cells, as well as among different nanoparticle concentrations (5, 10 and 25 µg) within the same treatment group. Data are presented as mean ± standard deviation (*n* = 3). One-way ANOVA followed by Tukey’s post hoc test was used for statistical analysis, where *P < 0.05, **P < 0.01, ***P < 0.001 and ****P < 0.0001 (scale bar represents 150 µm).

The biocompatibility of antibody-functionalized MNPs was evaluated using the Presto blue assay in MC3T3 cells and hADSCs. The results after 72 h of treatment (illustrated in Figure 6A–F) showed that TREK- and Piezo-functionalized MNPs exhibit enhanced metabolic activity at lower doses (5 and 10 µg), indicating their biocompatibility and potential to promote enhanced cellular metabolism. However, at a dose of 25 μg, there was a slight decrease in metabolic activity observed, probably due to moderate cytotoxicity or metabolic stress at higher dosages. Despite this reduction, the MNPs still exhibit good biocompatibility that is at least comparable to the control, and the MNPs are well tolerated by the cells.

Additionally, Live/Dead staining further confirms the biocompatibility of antibody-functionalized MNPs (Figure 6C and F). The predominance of green fluorescence in all images suggests that the majority of the cells are viable with excellent cell morphology, whereas minimal red fluorescence signifies a low percentage of dead cells. Overall, the findings have identified optimal and safe dosing of TREK- and Piezo-functionalized MNPs for mechanotransduction and osteogenic applications.

### Alkaline phosphate activity

**Figure 7:**
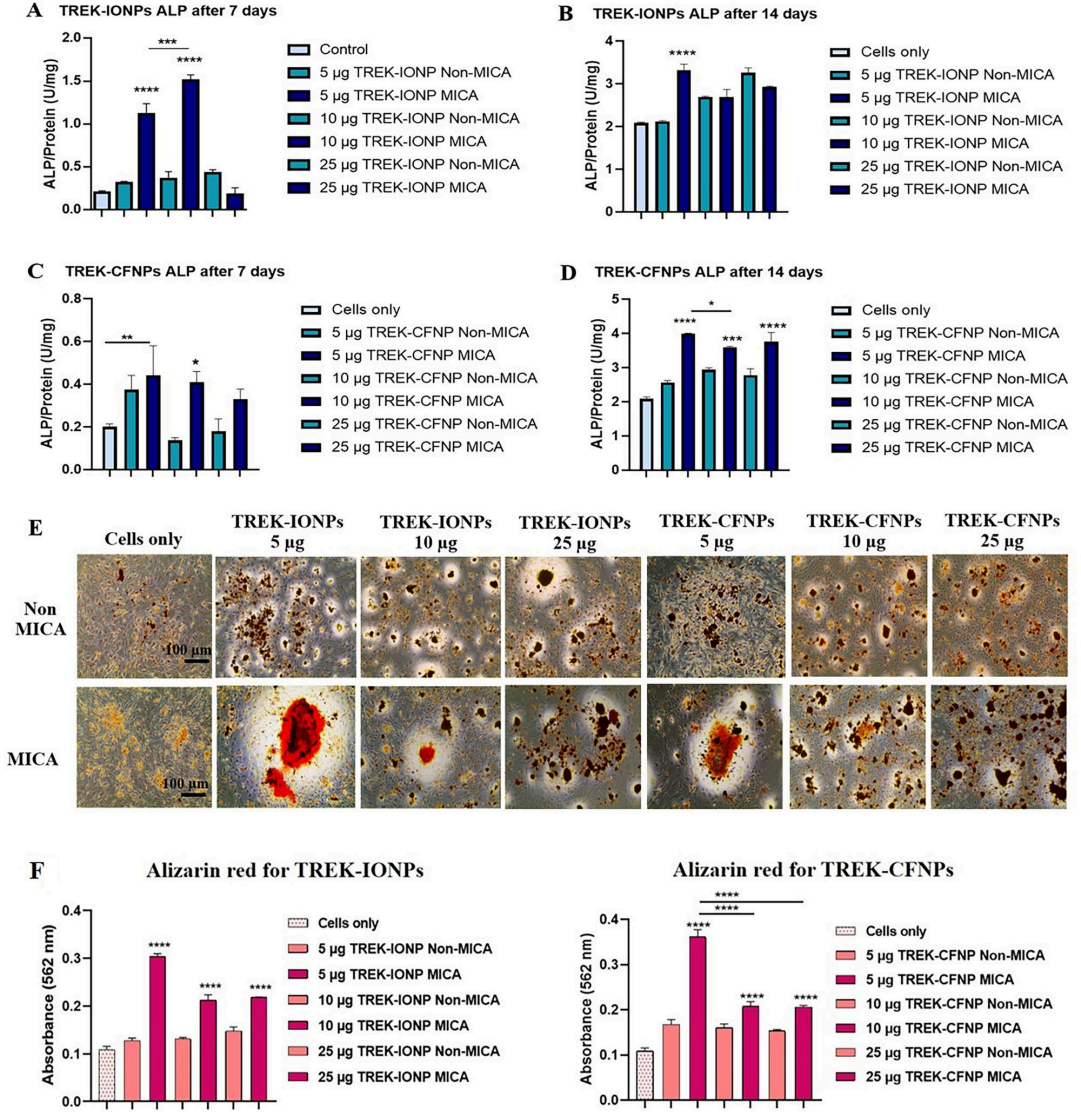
ALP enzymatic activity of MC3T3 cells after 7 and 14 days of MICA treatment using (**A**, **B**) TREK-IONPs and (**C**, **D**) TREK-CFNPs. (**E**, **F**) ARS optical images with quantitative analysis using CPC dye extraction after 21 days of treatment with and without MICA using TREK-IONPs and TREK-CFNPs in MC3T3 cells. Statistical comparisons were performed between MICA-treated and corresponding non-MICA-treated groups, as well as among different nanoparticle concentrations (5, 10 and 25 µg) within the same treatment group. Data are presented as mean ± standard deviation (*n* = 3). A two-way ANOVA test was used, and statistically significant differences were marked as * for *P* < 0.05, ** for *P* < 0.01, *** for *P* < 0.001 and **** for *P* < 0.0001 (scale bar: 100 µm).

**Figure 8:**
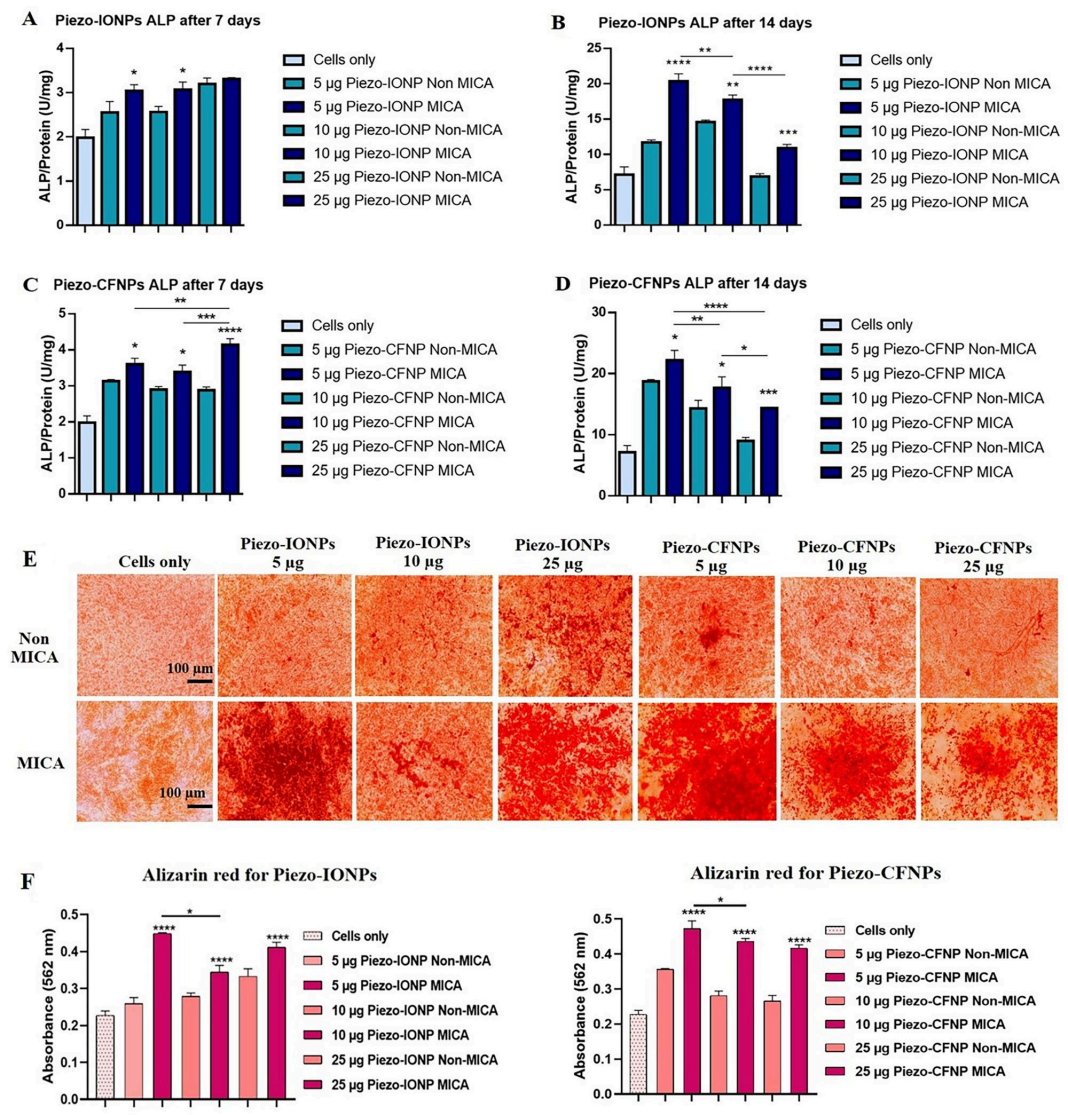
ALP enzymatic activity of hADSCs after 7 and 14 days of MICA treatment using (**A**, **B**) Piezo-IONPs and (**C**, **D**) Piezo-CFNPs. (**E**, **F**) ARS optical images with quantitative analysis using CPC dye extraction after 21 days of treatment with and without MICA using Piezo-IONPs and Piezo-CFNPs in hADSCs. Statistical comparisons were performed between MICA-treated and corresponding non-MICA-treated groups, as well as among different nanoparticle concentrations (5, 10 and 25 µg) within the same treatment group. Data are presented as mean ± standard deviation (*n* = 3). A two-way ANOVA test was used, and statistically significant differences were marked as * for *P* < 0.05, ** for *P* < 0.01, *** for *P* < 0.001 and **** for *P* < 0.0001 (scale bar: 100 µm).

To explore the osteogenic potential of TREK- and Piezo-functionalized MNPs under MICA, ALP activity was measured in both MC3T3 cells and hADSCs (Figures 7A–D and 8A–D). ALP, an important early-stage osteogenic marker, plays a crucial role in bone matrix mineralization. Colorimetric ALP analysis suggested that the ALP activity of MICA-treated groups is remarkably higher as compared to the non-MICA-treated groups from 7 to 14 days. Notably, MNP-treated groups at the lowest dose of 5 µg showed significantly higher ALP activity in both cell lines as compared to higher-dose groups.

### Alizarin Red staining

ARS was used to assess the mineralization of calcified nodules in MC3T3 cells and hADSCs after 21 days of treatment with and without MICA stimulation. As illustrated in Figures 7E and F and 8E and F, the red staining signifies calcium phosphate accumulation in the matrix around MC3T3 cells and hADSCs. The data revealed that the MICA-treated groups demonstrated considerably higher mineralization than the non-MICA groups after 21 days of treatment. Importantly, the lowest dose of 5 µg showed enhanced mineralization in both cell types as compared to higher doses. Quantitative analysis of the extracted dye using CPC confirmed these observations by showing a high absorbance value in CFNP-treated groups. Overall, these findings combined with ALP results indicated that CFNPs and IONPs under MICA stimulation offer a more effective osteoinductive platform for bone regeneration compared to non-MICA groups, while CFNPs provide superior magnetic responsiveness, particularly at low, biocompatible dosages.

## Discussion

Our previous studies have assessed the suitability of bacterially synthesized zinc- and cobalt-doped magnetite nanoparticles for biomedical applications. Specifically, we found that the moderate levels of cobalt required for optimum magnetic properties did not alter their cytotoxicity or affect osteogenic differentiation of the stem cells investigated [34]. Thus, despite the known cytotoxicity of cobalt ions, these results suggest that IONPs can be doped to sufficiently tailor their magnetic properties without compromising cellular biocompatibility.

Furthermore, PAA coating and antibody functionalization reduce MNPs degradation and potentially cobalt leaching, which increases biocompatibility. In support of this, cobalt core-based MNP (CFNP) in this study displayed negligible effects on cellular cytotoxicity and was comparable in their properties to IONP.

In this study, we used IONPs and CFNPs combined with external magnetic fields to induce MNP torque and remote activation of osteogenic signaling pathways. In our previous work, we have demonstrated the potential for iron-oxide MNP to target ion channels and drive osteogenesis in combination with MICA [3, 17, 35]. The results in the current study demonstrate the translatability of the MICA platform to other types of MNPs of varying sizes and coating concentrations. We demonstrate how cell differentiation can be achieved through the same mechanosensitive channels. Interestingly, a 5-μg MNP dose of either TREK- or Piezo-targeted MNP achieved optimal osteogenesis. While higher MNP doses also induced osteogenesis, the effects were less pronounced compared to the lower dose. This suggests that MNP aggregation at higher doses may induce steric hindrance that affects target channel binding. Indeed, our SEM/EDAX analysis confirmed effective MNP binding to the cells with minimal clustering at a 5-μg dose. In addition, we saw increased metabolic activity in both cell types in response to the 5-µg MNP dose. Increased metabolic activity or cell proliferation can also positively impact osteogenesis and may explain the increased effect at this dose. In our previous studies on MNP-mediated mechanotransduction, we used comparable doses (≈ 5 μg) to those in this study [3]. The results presented here validate this work and show that MNP doses need to be titrated to achieve the desired biological outcome for tissue engineering.

Our findings also show that both IONP and CFNP-based MNPs have overall comparable efficacy for activating both Piezo and TREK channels, with similar effects on osteogenesis. This suggests that both channels play a role in mechanotransduction and osteogenesis in both hADSCs and mouse osteoblasts (MC3T3). These results were observed despite IONP and CFNP showing differing levels of antibody functionalization. This suggests that the antibody density on the MNP surface is not significantly affecting signaling behavior in these cells, indicating a threshold level of antibody functionalization has been achieved. In addition, in previous reports using 250-nm particles, we have estimated coating concentrations of 10 and 20 μg of primary and secondary antibodies per mg of MNP, and in this report, we tested a range of binding of up to a maximum of 10 μg and 20 μg of primary and secondary antibodies per mg of MNP, respectively. Here, we show the 10:20 μg ratio of primary–secondary antibody concentration to be most effective in inducing a cellular response. In support of this, previous work from our group has shown that reaching a threshold density of ligands on the surface of MNP is required to achieve elevated signaling activity [22].

While the current study focused on targeted mechanostimulation of Piezo and TREK independently, it should be noted that other groups have established a link between Piezo and TREK signaling. Glogowska *et al.* recently demonstrated that Piezo channels can regulate mechanosensitivity of TREK in mouse fibroblasts with roles in wound healing [36]. Altogether, our current study emphasizes the importance of nanoparticle core composition and dose optimization for regulating osteogenic cell differentiation. This work highlights the potential of antibody-functionalized IONPs and novel CFNPs as a non-invasive strategy for targeted injectable bone tissue engineering therapies.

## Conclusions and future perspective

This study offers a comparative assessment of antibody-functionalized IONPs and CFNPs for stimulating osteogenesis via targeted activation of MS ion channels under MICA using novel MNPs. Both the MNPs were synthesized using the thermal decomposition method and functionalized with PAA via the ligand exchange method to enable stable aqueous dispersion and facilitate successful conjugation with TREK and Piezo antibodies, which are two essential MS ion channels in bone regeneration. The successful attachment of antibodies is validated by the shift in zeta potential measurements from previously PAA-functionalized MNPs. The SEM/EDAX analysis confirmed the effective cellular binding with minimal clustering at an optimal 5-µg dose. *In vitro* biological assays studied on MC3T3 and hADSCs revealed that CFNPs exhibit comparable performance to IONPs in key osteogenic parameters, including metabolic activity, ALP expression, and calcium mineralization, particularly at the minimal tested dosage of 5 µg. This indicates that greater doses may produce nanoparticle aggregation and steric hindrance, limiting effective target-channel interactions. The use of CFNPs for mechanoactivation applications is attractive due to high magnetic saturation, which in theory provides greater torque on MNPs and thus more effective mechanical stimulation of membrane-bound TREK and Piezo channels. This mechanotransduction activated specific downstream biochemical pathways that enhanced osteogenic differentiation and calcium deposition. Overall, these findings validate CFNPs as a more potent nanoplatform for magnetically stimulated MS ion channel in bone regeneration and emphasize the importance of nanoparticle composition and dose optimization. This work highlights the potential of antibody-functionalized IONPs and CFNPs as a promising, non-invasive strategy for targeted bone tissue engineering therapies. In the future, comprehensive *in vivo* investigations will be undertaken to evaluate the efficacy, biodistribution, and safety profile of these functionalized nanoparticles in bone regeneration models, facilitating translational applications in targeted, non-invasive bone tissue engineering. Additionally, we plan to explore the synergistic potential of dual antibody-functionalized IONPs and CFNPs to simultaneously activate multiple MS ion channels within a single nanoplatform under MICA.

## Supplementary Material

rbag145_Supplementary_Data

## Data Availability

The original contributions presented in the study are included in the article; further inquiries can be directed to the corresponding author.
