# Supplementary material for: Magnetic nanoparticle formulations to activate TREK and Piezo mechanosensitive ion channels (MICA) for enhanced osteogenesis
Source: Regen Biomater. 2026 Jul 15;13:rbag145. doi: 10.1093/rb/rbag145 (PMC13472695; doi:10.1093/rb/rbag145)
Supplement: Supplementary file 1 [file rbag145_supplementary_data.docx]

**SUPPORTING INFORMATION**

**Magnetic nanoparticle formulations to activate TREK1 and Piezo1 mechanosensitive ion channels (MICA) for enhanced osteogenesis**

**Kritika^1,2,*^,** **Michael Rotherham^1^, Yuxuan Du^1^, Kantawat Leerattanapetch^1^, Thomas J. Broomhall^1^, Indrajit Roy^2,3^ and Alicia J. El Haj^1,*^**

*^1^**Healthcare Technologies Institute, Institute of Translation Medicine, School of Chemical Engineering, University of Birmingham, Edgbaston, Birmingham B15 2TT, United Kingdom.*

*^2^Department of Chemistry, University of Delhi, Delhi-110007, India.*

*^3^Institute of Nano Medical Sciences, University of Delhi, Delhi-110007, India.*

Correspondence

***Prof. Alicia El Haj**

Email: a.elhaj@bham.ac.uk

***Dr. Kritika**

Email: [kritikav303@gmail.com](mailto:kritikav303@gmail.com)

**Table S1** Different antibody coating concentrations on anti-TREK1 MNPs using three different levels of goat anti-rabbit secondary antibody and TREK1 primary antibody conjugated to the nanoparticle surface. Abbreviation: T1, TREK1

| ***Condition No.*** | ***Anti-rabbit Secondary antibody* (μg/ml)** | ***TREK1 Primary antibody* (μg/ml)** |
| --- | --- | --- |
| **T1-1** | **0** | **0** |
| **T1-2** | **0** | **5** |
| **T1-3** | **0** | **10** |
| **T1-4** | **10** | **0** |
| **T1-5** | **10** | **5** |
| **T1-6** | **10** | **10** |
| **T1-7** | **20** | **0** |
| **T1-8** | **20** | **5** |
| **T1-9** | **20** | **10** |

**Figure S1** Bar graph comparing fold induction across antibody-coating conditions. (*p < 0.05) †All conditions were performed in triplicate and normalised to dsDNA quantification. Abbreviation: NC = negative control; SS = Sum of Squares; df = Degrees of Freedom; MS = Mean Square; F = F-statistic

**Figure S2** Comparison between the TREK1 primary antibody coating concentrations of 0, 5, and 10 µg/mL. (A) Table showing mean fold induction (FI) for each TREK1 primary antibody concentration and corresponding t-test p-values. (B) Bar graph comparing fold induction across TREK1 primary antibody concentrations. (*p < 0.05) Abbreviation: Primary Ab = TREK1 primary antibody.

**Figure S3** ICP-OES quantification of Fe release from MNPs (2.5 µg/cm²) with and without TREK functionalisation in PBS (pH 7.4) after 24 hr incubation at 37°C. Data are presented as mean ± standard deviation (n=3).
